# Semen collection and ejaculate characteristics of the Leopard Tortoise (*Stigmochelys pardalis*)

**DOI:** 10.1093/conphys/cox062

**Published:** 2017-11-09

**Authors:** Dawn M Zimmerman, Mark A Mitchell

**Affiliations:** 1 Smithsonian Conservation Biology Institute, National Zoological Park, 3001 Connecticut Avenue NW, Washington, DC 20013, USA; 2 Louisiana State University, Veterinary Teaching Hospital, 1909 Skip Bertman Drive, Baton Rouge, LA 70803, USA

**Keywords:** Leopard tortoise, *Stigmochelys pardalis*, semen collection, ejaculate characteristics, electroejaculation

## Abstract

The preservation of spermatozoa is an important tool used in conservation programs to increase the genetic diversity of threatened and endangered species. Although routinely used to manage conservation programs for higher vertebrates, there have been limited attempts to establish reproductive assistance programs for tortoises. The purpose of this study was to develop a model for collecting and characterizing semen in Testudinidae. Semen was collected from 13/16 (81.2%, 95% CI: 62–100) adult male leopard tortoises (*Stigmochelys pardalis*) via electroejaculation under propofol anesthesia. Semen samples were collected most frequently after the second series of electrostimulations (6/13, 46.1%), with fewer animals producing semen after the first (5/13, 38.5%) or third (2/13, 15.4%) electrostimulations. The average volume of a semen sample in the tortoises was 0.26 ml (standard deviation: 0.16, minimum–maximum: 0.1–0.6), the average spermatozoal concentration was 101.62 × 10^6^/ml, and the average motility at time of collection was 57.3%. A rapid decrease in motility was observed in refrigerated samples over 24 h resulting in a median motility of 0% at 24 h post-collection. The results of this study suggest that electroejaculation is a safe and efficient method for collecting semen from leopard tortoises.

## Teaser

Chelonians are one of the most threatened groups of vertebrates in the world and conservation attempts are limited by a lack of reproductive assistance programs. This study developed a safe protocol for semen collection via electroejaculation of leopard tortoises, as a tool for use in chelonian reproductive assistance programs.

## Introduction

To date, ~335 species of chelonians from the phylogenetic order Testudines have been identified (van Dijk *et al.*, 2014). Of these species, 107 (31.9%) are Critically Endangered [CR] or Endangered [EN]; this number increases to 167 (49.9%) for threatened species inclusive of CR, EN and vulnerable [VU] populations, and to 175 (52.2%) species when comprising threatened and recently extinct populations (IUCN, 2010; van Dijk *et al.*, 2014). By estimating threat rates for data-deficient species and including recently extinct species, more than half of all modern chelonian species are either threatened or extinct—a number that has increased substantially over the years and makes chelonians one of the most endangered groups of vertebrates. Threats to chelonians include habitat destruction and being victims of bycatch, unsustainable collection for meat, poaching for medicinal value, and the pet and tortoise shell trade (IUCN, 2010). Particularly notable is the poaching of Asian chelonians, with 17 (68%) of the top 25 endangered turtles found in Asia (IUCN, 2010). Based on the risks facing this group and the ongoing pressures they face, the significance and need of conserving chelonian species is apparent.

Protection efforts are being made for chelonians, including environmental and regulatory initiatives, as well as more pro-active methods such as head-start programs that attempt to offset population declines by supplementing wild populations with animals propagated in captive breeding programs. However, some of these strategies are not always feasible (e.g. largescale protection of environments, successful natural breeding of captive populations), are poorly enforced (e.g. anti-poaching laws), or have inherent risks associated with them (e.g. introduction of pathogens into the wild). Assisted reproduction techniques offer conservationists the ability to enhance the production of a species while sustaining or increasing genetic diversity. Semen collection and cryopreservation provide an effective method for preserving germplasm and genetic diversity of a species, and may well be the most effectual method for conservation of a species (Gee, 1984). This is especially useful for animals with a limited reproductive period or that exhibit seasonal spermatogenesis, such as chelonians. Validation of germplasm collection, storage and artificial insemination techniques is warranted for chelonians; however, few methods describing semen collection have been reported for these species.

While limited in comparison to reports in mammalian species, successful antemortem semen collection in different species of reptiles has been documented (Table 1). Manual stimulation techniques have been described to facilitate hemipene erection and ejaculation in snakes (Mengden *et al.*, 1980; Samour, 1986; Fahrig *et al.*, 2007; Tourmente *et al.*, 2007; Zacariotti *et al.*, 2007) and some lizards (Todd, 2003; Molinia *et al.*, 2010); however, these samples were often found to be contaminated with eliminated urates, faeces and anal gland secretions (Durrant, 1990; Tourmente *et al.*, 2007). In chelonians, because of the presence of the shell, electroejaculation remains the only method for semen collection (Millar and Watson, 2001).

**Table 1:** Antemortem-collected semen analysis data from reptilian species

Species	Sample size (*n*)	Collection method	Volume (ml; median or range)	Concentration (mean; × 10^6^ spermatozoa/ml)	Motility (mean or range)	Reference
**Squamates**						
Checkered garter snake (*Thamnophis marcianus*)	4	EE	0.05–0.1	Not numerically reported	50–70%	Quinn *et al.* (1989)
Angolan python (*Python anchietae*)		Manual	0.1–0.4	1500		Mengden *et al.* (1980)
Timor python (*Python timoriensis*)		Manual	0.1–0.4	1500		Mengden *et al.* (1980)
Sinaloan milk snake (*Lampropeltis triangulum sinaloae*)	Not reported	Manual	0.25–0.5	>1000 (estimate)	Not reported	Samour (1986)
Black rat snake (*Elaphe obsoleta obsoleta*)	Not reported	Manual	Not reported	Not reported	Not reported	Samour (1986)
Brazilian rattlesnake (*Crotalus durissus terrificus*)	28	Manual (lidocaine)	0.02	1380	63.9%	Zacariotti *et al.* (2007)
Corn snake (*Elaphe guttata*)	22	Manual	0.01	852	92.5%	Fahrig *et al.* (2007)
Corn snake (*Elaphe gutatta*)	5	Manual	0.002–0.005	9.7	91.9%	Mattson *et al.* (2007)
Argentine boa constrictor (*Boa constrictor occidentalis*)	7	Manual	Not reported	Not reported	63%^a^	Tourmente *et al.* (2007)
**Lizards**						
McCann’s Skink (*Oligosoma maccanni)*	Not reported	Manual	Not reported	Not reported	Not reported	Molinia *et al.* (2010)
Green Iguana	16	EE	0.05	269	78%	Zimmerman *et al.* (2013)
Chelonians						
Green sea turtle (*Chelonia mydas*)	4	EE	5–27	110–2930	1–80%	Platz *et al.* (1980)
Red-eared pond turtle (*Trachemys scripta elegans*)	1	EE	0.2	200	85%	Platz *et al.* (1980)
Galapagos tortoise (*Chelonoidis nigra*)	3	EE	3.2–75	240–655	40–80%	Platz *et al.* (1980)
Green sea turtle (*Chelonia mydas*)	28	EE	Not reported	470	36%	Wood *et al.* (1982)
Madagascar ploughshare tortoise (*Astrochelys yniphora*)	1	EE	Not reported	Not reported	Not reported	Juvik *et al.* (1991)
Olive ridley turtle (*Lepidochelys olivacea*)	6	EE	1 (0.01–2.2)	67.3 (11.5–150)	28.25% (0–98%)	Tanasanti *et al.* (2009)
Hawksbill turtle (*Eretmocheyls imbricata*)	1	EE	4.4	512	60%	Tanasanti *et al.* (2009)
Black marsh turtle (*Siebenrockiella crassicollis*)	9	EE	Not reported	Not reported	Not reported	Kimskulvech and Suttiyotin (2012)
Hawksbill turtle (*Eretmocheyls imbricata*)	14 (2 animals)	EE	0.5 (0.2–1.5)	325 (100–645)	2–54%	Kawazu *et al.* (2014)
					2.5–39.5%	
**Crocodilians**						
Broad-nosed caiman (*Caiman latirostris*)	7	Manual	<0.1	522	78.5%	Larsen *et al.* (1992)
Australian saltwater crocodile (*Crocodylus porosus*)	30 (24 animals)	Manual	0.91 ± 0.16	2290 ± 260	50.7 ± 4.2	Johnston *et al.* (2014)

^a^Analysis after dilution at 50% in phosphate-buffered saline (PBS).

To date, semen collection has been performed via electroejaculation in four species of turtles and two species of tortoises (Table 1), including green sea turtles (*Chelonia mydas*, Platz *et al.*, 1980; Wood *et al.*, 1982), olive ridley turtles (*Lepidochelys olivacea*, Tanasanti *et al.*, 2009), a red-eared slider turtle (*Trachemys scripta elegans*, Platz *et al.*, 1980), hawksbill turtles (*Eretmochelys imbricata*, Tanasanti *et al.*, 2009; Kawazu *et al.*, 2014), Galapagos tortoises (*Testudo elephantopus*, Platz *et al.*, 1980) and a ploughshare tortoise (*Geochelone yniphora*, Juvik *et al.*, 1991). Semen evaluation, including volume and sperm concentration, motility and morphology, has been reported in hawksbill turtles (Tanasanti *et al.*, 2009; Kawazu *et al.*, 2014), olive ridley turtles (Tanasanti *et al.*, 2009), Galapagos tortoises (Platz *et al.*, 1980), green sea turtles (Platz *et al.*, 1980) and a red-eared slider turtle (Platz *et al.*, 1980); however, storage methods have not been described except in a single study evaluating extenders in olive ridley turtles (Sirinarumitr *et al.*, 2009). These examples represent a small proportion (1.8%) of all chelonians and reinforce a need for additional study to develop functional assisted reproduction programs for these vulnerable animals.

The purpose of this study was to: (i) develop a successful method for semen collection in Testudinidae using the leopard tortoise (*Stigmochelys pardalis*) as a model, (ii) characterize leopard tortoise semen parameters including appearance, volume, concentration, sperm motility and morphology; and (iii) determine the survival of tortoise sperm at 4°C. The specific hypotheses being tested in this study were: (i) that semen could be collected safely and consistently via electroejaculation and (ii) sperm viability would last 24 h at standard refrigeration temperature (4°C, 39.2°F).

## Materials and methods

This study was performed in accordance with the regulations set forth by the University of Illinois Institutional Animal Care and Use Committee (protocol: 08-128). Sixteen adult male wild-caught leopard tortoises from a private breeding collection in El Salvador were used for this study. This study was completed in June, during the breeding season for leopard tortoises (May through October; Ernst and Barbour, 1989).

**Figure 1:**
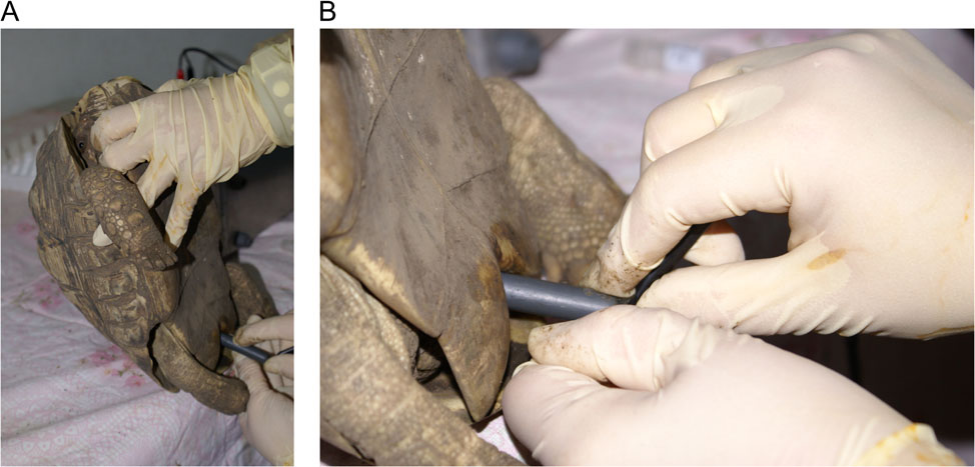
(**A**) Electroejaculation in a leopard tortoise. The tortoises were held upright to reduce any pressure on their lungs. (**B**) Note the depth to which the probe could be inserted

**Figure 2:**
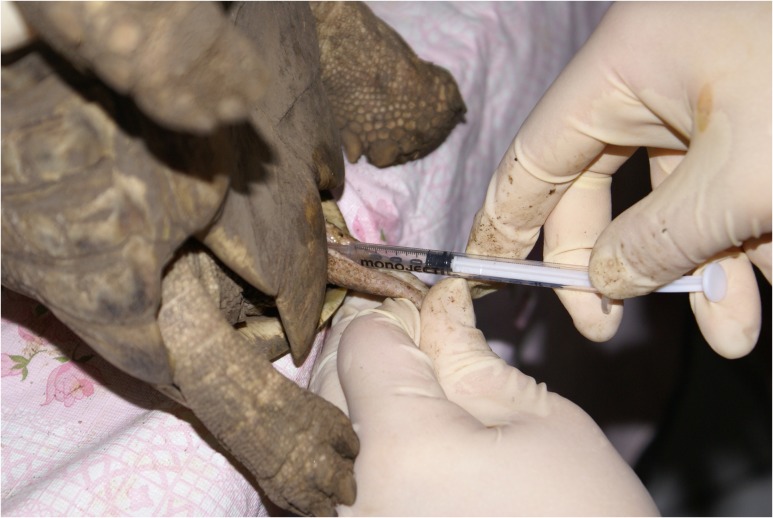
The ejaculate was collected using a 1 mL syringe

A thorough physical examination was performed on each tortoise to ensure it was in good health, and a body weight was obtained for each animal. To minimize discomfort during the electroejaculation procedure, the tortoises were anesthetized with propofol (Propoflo™, Abbott Animal Health, Chicago, IL, USA; 10 mg/kg) intravenously into the subcarapacial sinus using a 22-gauge 1.5-inch needle. During the procedure, the tortoises were held up right to reduce any pressure on their lungs. To avoid contamination of the semen, the cloaca was lavaged with 20 ml physiological saline (0.9%) prior to sample collection. The electroejaculator consisted of a variable voltage power source and a plastic rectal probe (2.6 cm diameter) with three 3.0 mm longitudinal electrodes (Carol C. Platz, Sandy, OR, USA). Electroejaculation was performed using 15 three-second cloacal intromissions at 4 V, followed by 15 three-second additional cloacal intromissions at 6 V (Fig. 1). The lubricated probe was inserted to the approximate level of the kidneys with the electrodes directed dorsally. As stimulation was applied, the probe was retracted caudally, presumptively producing contractions of the vas deferens and movement of semen towards the dorsal penile groove to the cloacal opening. Tortoises were electroejaculated up to three times using this protocol, and a 3-min break was given in between each electroejaculation series. Once a successful ejaculate was collected, the process was discontinued and the number of times electroejaculation was required to obtain semen was recorded. The semen was collected using a 1 ml syringe without a needle (Fig. 2).

Once collected, the ejaculates from each animal were evaluated for volume and colour, as well as spermatozoal concentration, motility and morphology. Volume was measured using the 1 mL collection syringe. Each fresh semen sample was extended by 1:10 dilution with modified Ham’s F10 with albumin (Irvine Scientific, Santa Ana, CA, USA). Motility of the spermatozoa in the extended semen was estimated by placing a drop of the sample on a slide under a coverslip at ambient temperature and estimating the percentage of progressively motile sperm cells to the nearest 5% in five different microscopic fields under ×400 magnification. To evaluate concentration, the extended semen was diluted 1:10 in formol buffered saline for a total dilution of 1:100, and sperm cells were counted in a hemocytometer chamber under phase contrast microscopy (×400). Semen concentration of the sample was calculated with the hemocytometer sperm count and a conversion factor, taking into account both dilutions. Morphology was also evaluated, by observing 100 sperm cells under oil-immersion microscopy (×1000). The percentage of each morphologic abnormality was determined for each sample.

The semen collected from the tortoises was evaluated for viability following refrigerated storage. The diluted semen was held at room temperature for ~10 min while the initial semen evaluation was performed. The extended semen was then diluted 1:1 with ambient-temperature Refrigeration Test Yolk Buffer (Irvine Scientific, Santa Ana, CA, USA). A 0.3 ml aliquot of the diluted sample was pipetted into a 2 ml cryotube (Nunc, Rochester, NY, USA). Motility of the sperm (0 h) was estimated before cooling. The rate of forward progression was not evaluated. The vial was placed in a standard refrigerator (4°C) within a styrofoam rack. To determine sperm longevity, motility was estimated every 24 h until motility dropped to 0%.

The distribution of the data for ejaculate volume, motility and spermatozoal concentration were evaluated separately using the Shapiro–Wilk test, skewness, kurtosis and q–q plots. For normally distributed values, the mean, standard deviation (SD) and minimum/maximum (min–max) were calculated. For non-normally distributed values, the median, 25–75% quartiles and min–max were calculated. Pearson’s correlation test was used to determine if body weight was correlated to spermatozoal count, and whether motility was correlated to sample volume or spermatozoal count. Levene’s test for equality of variances was used to determine if the data was homogeneous. One-way ANOVA was used to assess between group differences for ejaculate volume, motility and spermatozoal concentration by ejaculate appearance for normally distributed data. For data that were not normally distributed, a Kruskal–Wallis test was used. Motility in the different cooling methods was evaluated over time using Friedman’s test for non-parametric analysis of repeated data. Where differences were apparent, Friedman’s tests were used to compare spermatozoal motility at the time of collection (time 0) to the samples after 24, 48 and 72 h of refrigeration. Values of *P* ≤ 0.05 were considered statistically significant. Statistical analyses were performed using SPSS 22.0 (IBM Inc., Armonk, NY, USA).

## Results

The ambient daytime temperature range during the time of sampling was 29–32°C (84–90°F). The mean weight of the tortoises used in this study was 3.2 kg (SD: 0.4, min–max: 2.5–4.0 kg). The average induction volume of propofol was 3.2 ml (SD: 0.4, min–max: 2.5–4.0 ml). The median time to achieve sedation was 3.2 min (SD: 1.42, min–max: 1.0–6.0). Semen was successfully collected from 13 (81.2%, 95% CI: 60–100) of the 16 study tortoises. The majority of the tortoises produced a semen sample after the second series of electrostimulations (6/13, 46.1%), with fewer animals producing semen after the first (5/13, 38.4%) or third (2/13, 15.4%) series of electrostimulations. Significant muscle contractions and hind leg extension were noted during several series of the electrostimulations. Eversion of the penis was not observed prior to ejaculation, and is therefore not considered necessary for semen collection following EE in leopard tortoises. The mean volume of semen collected from the tortoises was 0.26 ml (SD: 0.16, min–max: 0.1–0.6 ml; Table 2). The mean spermatozoal concentration of a semen sample was 101.62 × 10^6^/ml (SD: 91.43 × 10^6^/ml, min–max: 3.20 × 10^6^–314.7 × 10^6^/ml; Table 2). There was no significant correlation between spermatozoal concentration and body weight (*R*: −0.49, *P* = 0.09) or sample volume (*R*: 0.18, *P* = 0.55).

**Table 2:** Ejaculate characteristics of the Leopard Tortoise (*Stigmochelys pardalis*)

	Mean	Min	Max	SD
Volume (ml)	0.26	0.1	0.6	0.16
Concentration (×10^6^ Spermatozoa/ml)	101.62	3.2	314.7	91.43
Motility (%)	57.3	10	80	18.1

There were four different colour types noted for the semen samples: colourless, tan, white and blood-tinged. The majority of the samples collected were clear and colourless (8/13, 61.5%) in appearance, followed by tan (3/13, 23.1%), white (1/13, 7.7%) and blood-tinged (1/13, 7.7%). There was no significant difference in the volume of ejaculate based on the colour of the ejaculate (KW = 4.9, df = 4, *P* = 0.2), nor was there a significant difference in spermatozoal counts by ejaculate colour (*F* = 2.81, df = 4, *P* = 0.10).

The mean motility of the spermatozoa at the time of collection was 57.3% (SD: 18.1%, min–max: 10–80%; Table 2). There was no significant difference in motility based on ejaculate colour (*F* = 0.979, df = 4, *P* = 0.47). Motility was not correlated to spermatozoal count (*R*: 0.06, *P* = 0.83) or sample volume (*R*: 0.13, *P* = 0.66). The overwhelming majority of spermatozoa were normal in appearance (mean: 84.2%, SD: 16.5%, min–max: 40–100%). The most common spermatozoal anomaly observed was bent tails (mean: 12.6%, SD: 17.1%, minimum–maximum: 0–13%).

There was a significant reduction (*F* = 61.87, df = 3, *P* = 0.0001) in spermatozoal motility over time. Median motility at the time of sample collection was 60.0% (10–90%: 38.0–76.0, min–max: 10.0–80.0), and significant reductions in motility were noted from this starting point after 24 h (median: 0%, 10–90%:0–42.0%, min–max: 0–50%; *F* = 13, df = 1, *P* = 0.0001), 48 h (median: 0%, 10–90%:0–30.0%, min–max: 0–30%; *F* = 13, df = 1, *P* = 0.0001) and 72 h (median: 0%, 10–90%: 0–4.0%, min–max: 0–20.0%; *F* = 13,df = 1, *P* = 0.0001) of refrigeration.

Follow-up on the animals the next year found them all to be in good health with no known ill-effects or long-term complications from the electroejaculation procedure.

## Discussion

The present study suggests that electroejaculation is an effective means by which to safely collect semen from leopard tortoises; however, it is notable that semen could not be collected from three (19%) of the tortoises. All three tortoises were considered to be in good health based on their physical examinations and body weight; however, reptiles are excellent at masking disease and it is possible that there was an underlying issue that affected their ability to produce a semen sample. Other potential theories for why these three animals did not produce an ejaculate were anesthetic depth and recent breeding activity. Although the plane of anesthesia could have had an effect on the success of collection in these tortoises, as has been reported in mammals (see paragraph below), the authors felt it was necessary to use an anesthetic to minimize any discomfort from the procedure and tried to control for any affect by anesthetizing all 16 tortoises with the same dose of propofol. It is also possible, and more likely, that these tortoises had recently been copulating with female tortoises. The study subjects all came from an active breeding colony, and copulation was observed throughout the day in this colony.

It is not known if anesthesia and/or stress may affect semen quantity or quality in reptiles. Differences in response to electroejaculation procedures under various anesthetics have been reported in mammals, including the degree of penile protrusion and the electrical pulse stimulation necessary to achieve ejaculation, but did not affect sperm quantity or quality (Ibex spp., Santiago-Moreno *et al.*, 2011). In addition, lower ejaculate volume has been reported in anesthetized mammals when compared to collection in conscious mammals, but sperm concentration and quality were unaffected (chinchillas, Busso *et al.*, 2005). Interestingly, stress studies in non-domestic mammals have suggested that electroejaculation-induced cortisol secretion has not been shown to adversely affect semen quality or reproductive function (Wildt *et al.*, 1984). Electroejaculation has been performed in some chelonian species without anesthesia with no associated discomfort or trauma reported (Platz *et al.*, 1980); however, based on the findings of the current study, there certainly appears to be some discomfort (e.g. significant muscle contractions) associated with this procedure.

Gross examination of the semen samples included colour and volume. Semen volumes obtained from the leopard tortoises in this study averaged 0.26 ± 0.16 ml, an average volume consistently smaller than most chelonian samples collected via electroejaculation (0.5–4.4 ml) but comparable to the red-eared pond turtle (0.2 ml) (Platz *et al.*, 1980; Tanasanti *et al.*, 2009; Kawazu *et al.*, 2014). These volumes are also much smaller than those produced by mammals, but this was not unexpected because reptiles do not have accessory glands that produce the larger volume of fluid seen in mammalian ejaculates (Gist *et al.*, 2000; Zimmerman *et al.*, 2013). Considering the variation in ejaculate volumes collected within the same species (e.g. hawksbill turtle averaging 0.5 ml in one study (Kawazu *et al.*, 2014) and 4.4 ml in another (Tanasanti *et al.*, 2009), and large ranges (e.g. Galapagos 3.2–75 ml, Platz *et al.*, 1980); Table 1), individual variation may largely affect average volumes. In addition, it is possible that seminal volume may vary depending on the season collected, with higher volumes in autumn at the end of the spermatogenic period and in the spring before regression of the epididymis (Gist *et al.*, 2000) depending on the species and geographic region. Although not always reported, increased ejaculate volumes could also reflect urine contamination. Because of the clear to cloudy appearance of the ejaculates, and the presence of monosodium urate crystals in some samples, it was suspected that urine contamination did commonly occur in these samples—but was not reflected in high semen volumes. Interestingly, one report suggests that the presence of urine increased motility of sperm post-ejaculation in hawksbill turtles, either due to decreasing the viscosity and allowing for increased motility or due to activation under low pH conditions (Kawazu *et al.*, 2014). However, urine contamination of sperm in mammals is known to significantly decrease sperm motility and is a major cause of infertility (Griggers *et al.*, 2001), as has also been speculated in crocodilians (Clulow and Clulow, 2016).

The majority of ejaculates were clear/colourless, in contrast to some squamate and lizard studies in which a tan or white coloration was most often observed (Fahrig *et al.*, 2007; Zimmerman *et al.*, 2013). The white and tan samples in the squamates were found to be significantly more likely to have higher sperm counts than clear/colourless samples. In the current study, there was only one white coloured ejaculate, so it was not possible to make this same comparison. One tortoise sample was blood-tinged, and was most likely due to minor cloacal trauma—either from a pre-existing injury or from the probe during the EE procedure. Care should be taken to not apply excessive pressure during cloacal intromissions.

The average spermatozoal concentration (101.62 × 10^6^/ml) in the samples collected from the leopard tortoises was comparable to olive ridley (Tanasanti *et al.*, 2009) and red-eared pond turtles (Platz *et al.*, 1980), but trended lower than that observed in hawksbill turtles (Tanasanti *et al.*, 2009; Kawazu *et al.*, 2014), green sea turtles (Platz *et al.*, 1980; Wood *et al.*, 1982) and Galapagos tortoises (Platz *et al.*, 1980) (Table 1). This difference may be attributed to species differences, time of year during collection, breeding status or collection technique.

The average motility of the spermatozoa (57.3%) at the time of collection was within the range of the other chelonian species that have been examined (Table 1). While this percent motility is lower than that considered normal for many higher vertebrates (Ax *et al.*, 2000), it was sufficient for producing offspring in a garter snake (50% motility, 0.05 ml) following artificial insemination.

A significant reduction in motility was observed over time while the samples were refrigerated, with a median 0% motility being identified after 24 h of refrigeration. While more comparable to fish and amphibian spermatozoa that become immobile within minutes after isolation (Billard and Cosson, 1992) as well as avian and mammalian spermatozoa which lose motility within 24–48 h without extension (Ashizawa *et al.*, 1976; Cupps, 1987), the loss of motility in this study is in contrast to that reported with *Chrysemys picta* spermatozoa which remained over 70% viable for 40 days (Gist *et al.*, 2000). The extended viability of *C. picta* spermatozoa was surmised to be necessary for the often extended delay to fertilization within the female’s oviduct (Gist *et al.*, 2000), which is believed to also occur in the leopard tortoise. The difference in longevity of motile spermatozoa between these two chelonian studies could be due to the time (season) of collection, the electroejaculation process (versus post-mortem collection), or the species involved (Emydidae versus Testudinidae). It is possible that while the motility decreased rapidly with time, the viability was maintained and could have been activated/re-activated by dilution or avoided altogether with rapid washing of the spermatozoa to remove contaminants such as urates and to normalize osmolarity and pH; however, this deserves further study. Also important to note is that Gist *et al.* (2000) found motility of some turtle (*Sternotherus odoratus* and *C. picta*) spermatozoa to be higher at reduced temperatures (versus his hypothesis that the highest motility would be observed at ambient temperatures in these ectothermic species), and the authors correlated this finding to the observation that breeding in these species occurs when temperatures are decreasing. Ultimately, cryopreservation protocols also need to be established for chelonians to guarantee long-term storage and transport of these samples.

The only morphological abnormality observed in the leopard tortoise spermatozoa were bent tails, which was also the most common morphological anomaly observed in semen samples from green iguanas (Zimmerman *et al.*, 2013) and corn snakes (Fahrig *et al.*, 2007). Bent tails are considered an iatrogenic change, developing post-collection due to exposure to a hypotonic solution or rapid cooling during sample buffering or dilution (Barth and Oko, 1989). In this study, the semen was diluted with modified Ham’s F10 with albumin that was warmed to room temperature, and was unlikely to be the cause of such a morphological change. Changes in pH may also result in morphological aberrancies, commonly altered by urine contamination (Kawazu *et al.*, 2014). Unfortunately, sample pH was not measured in this study, but urine contamination was noted, so it is possible it played a role.

Data from this study indicate that leopard tortoise spermatozoa collected via EE should be of adequate quality to use for insemination. However, the rapid decrease in motility observed in these animals will limit the chances of a successful insemination to within the first 24 h of collection. It is possible that using a different dilution/storage agent might yield longer sperm survivability. Properties known to affect spermatozoal viability include temperature, osmolarity, and ionic and pH sensitivity (Gist *et al.*, 2000). However, it may be difficult to extrapolate from studies in mammalian and avian species due to physiological differences, e.g. *C. picta* spermatozoa were unresponsive to ionic concentrations that reportedly increase sperm motility in other species (Gist *et al.*, 2000). Various media have been used for buffering, diluting and preserving reptile spermatozoa (Mengden *et al.*, 1980; Larsen *et al.*, 1982; Gist *et al.*, 2000). Extenders found most suitable for maintaining viability of sea turtle semen include refrigeration medium test yolk buffer and Tyrode medium supplemented with albumin, lactate and pyruvate (Sirinarumitr *et al.*, 2009). The use of antibiotics in cryopreservation may be warranted to increase the longevity of the samples when stored long term.

Within reptilian species, successful AIs have only been reported in alligators and snakes with fresh semen (Watson, 1990; Mattson *et al.*, 2007). This is, in part, due to unsuccessful cryopreservation of reptilian spermatozoa. Attempts in the green turtle (using glycerol or DMSO extenders) yielded only 2% motility post-thaw (Platz *et al.*, 1980) and attempts preserving snake spermatozoa in various diluents was poor (Samour, 1986; Fahrig *et al.*, 2007). Platz *et al.* (1980) reported that the midpiece bundles of chelonian spermatozoa are fragile which may not allow for successful cryopreservation. Despite the current inability to preserve chelonian spermatozoa, there is a ‘large window of opportunity’ in which to inseminate females with collected sperm because of female chelonian’s ability to store viable sperm within their reproductive tract for prolonged periods of time, reportedly years (Gist and Jones, 1989).

In mammals, including humans, electroejaculation reportedly has few side-effects. Possible complications from electroejaculation procedures in reptiles have only been reported once in a ploughshare tortoise that subsequently died from suspected renal failure (Juvik *et al.*, 1991); although no timeline, gross necropsy or histopathology findings could confirm/substantiate a diagnosis of renal failure nor link any renal pathology to the electroejaculation procedure. However, damage from electrical stimulations to susceptible organs remains a concern, especially of the kidneys due to their proximity to the testes in reptile species. That said, no other known complications or concerns have been reported in other reptile species. In this study, all sampled tortoises were observed the following year and appeared to be in good health with no known ill-effects/long-term complications from the EE procedure.

Generalized conclusions from this study can be offered; however, continued research of semen collection techniques and analysis is necessary due to the variability between chelonian genera. Future efforts in characterization of tortoise electroejaculates should include imaging of spermatozoa, live/dead stains and measured pH, osmolality, and rates of progression, which unfortunately could not be assessed in this study since performed in field conditions. Further research into the effect, if any, of anesthesia on semen collection and parameters might prove useful. It is useful to note that differences in spermatozoal quantity and quality may well be significant between species; previous studies on semen analysis on snakes indicate wide interspecies and intraspecies (captive versus wild) variation and it has been suggested that reference values for seminal characteristics cannot be established across species (Fahrig *et al.*, 2007; Zacariotti *et al.*, 2007). Additional studies are also needed to develop storage protocols and artificial insemination techniques in an effort to establish reproductive assistance programs for these species. The advent of gamete cryopreservation and artificial insemination techniques will confer great potential for genetic conservation in chelonians. Once established, cryopreservation protocols can help to conserve germplasm from underrepresented captive individuals as well as allow for the translocation of genetics between wild populations and into captive populations (Durrant, 1990).
